# Enhanced Statistical Tests for GWAS in Admixed Populations: Assessment using African Americans from CARe and a Breast Cancer Consortium

**DOI:** 10.1371/journal.pgen.1001371

**Published:** 2011-04-21

**Authors:** Bogdan Pasaniuc, Noah Zaitlen, Guillaume Lettre, Gary K. Chen, Arti Tandon, W. H. Linda Kao, Ingo Ruczinski, Myriam Fornage, David S. Siscovick, Xiaofeng Zhu, Emma Larkin, Leslie A. Lange, L. Adrienne Cupples, Qiong Yang, Ermeg L. Akylbekova, Solomon K. Musani, Jasmin Divers, Joe Mychaleckyj, Mingyao Li, George J. Papanicolaou, Robert C. Millikan, Christine B. Ambrosone, Esther M. John, Leslie Bernstein, Wei Zheng, Jennifer J. Hu, Regina G. Ziegler, Sarah J. Nyante, Elisa V. Bandera, Sue A. Ingles, Michael F. Press, Stephen J. Chanock, Sandra L. Deming, Jorge L. Rodriguez-Gil, Cameron D. Palmer, Sarah Buxbaum, Lynette Ekunwe, Joel N. Hirschhorn, Brian E. Henderson, Simon Myers, Christopher A. Haiman, David Reich, Nick Patterson, James G. Wilson, Alkes L. Price

**Affiliations:** 1 Department of Epidemiology, Harvard School of Public Health, Boston, Massachusetts, United States of America; 2 Department of Biostatistics, Harvard School of Public Health, Boston, Massachusetts, United States of America; 3 Broad Institute of Harvard and Massachusetts Institute of Technology (MIT), Cambridge, Massachusetts, United States of America; 4 Montreal Heart Institute, Montréal, Canada; 5 Département de Médecine, Université de Montréal, Montréal, Canada; 6 Department of Preventive Medicine, University of Southern California Keck School of Medicine, Los Angeles, California, United States of America; 7 Department of Genetics, Harvard Medical School, Boston, Massachusetts, United States of America; 8 Department of Epidemiology, Johns Hopkins University, Baltimore, Maryland, United States of America; 9 Welch Center for Prevention, Epidemiology and Clinical Research, Johns Hopkins University, Baltimore, Maryland, United States of America; 10 Department of Biostatistics, Bloomberg School of Public Health, Johns Hopkins University, Baltimore, Maryland, United States of America; 11 Institute of Molecular Medicine and Division of Epidemiology, School of Public Health, University of Texas Health Sciences Center at Houston, Houston, Texas, United States of America; 12 Departments of Medicine and Epidemiology, University of Washington, Seattle, Washington, United States of America; 13 Cardiovascular Health Research Unit, University of Washington, Seattle, Washington, United States of America; 14 Department of Epidemiology and Biostatistics, School of Medicine, Case Western Reserve University, Cleveland, Ohio, United States of America; 15 Department of Medicine, Division of Allergy, Pulmonary and Critical Care, Vanderbilt University, Nashville, Tennessee, United States of America; 16 Department of Genetics, University of North Carolina, Chapel Hill, North Carolina, United States of America; 17 Department of Biostatistics and Epidemiology, Boston University School of Public Health, Boston, Massachusetts, United States of America; 18 Jackson Heart Study, Jackson State University, Jackson, Mississippi, United States of America; 19 University of Mississippi Medical Center, Jackson, Mississippi, United States of America; 20 Department of Biostatistical Sciences, Division of Public Health Sciences, Wake Forest University, Winston-Salem, North Carolina, United States of America; 21 Center for Public Health Genomics, University of Virginia, Charlottesville, Virginia, United States of America; 22 Department of Biostatistics and Epidemiology, University of Pennsylvania, Philadelphia, Pennsylvania, United States of America; 23 National Heart, Lung, and Blood Institute (NHLBI), Division of Cardiovascular Sciences, National Institutes of Health, Bethesda, Maryland, United States of America; 24 Department of Epidemiology, Gillings School of Global Public Health Chapel Hill, North Carolina, United States of America; 25 Lineberger Comprehensive Cancer Center, University of North Carolina, Chapel Hill, North Carolina, United States of America; 26 Department of Cancer Prevention and Control, Roswell Park Cancer Institute, Buffalo, New York, United States of America; 27 Northern California Cancer Center, Fremont, California, United States of America; 28 Stanford University School of Medicine and Stanford Cancer Center, Stanford, California, United States of America; 29 Division of Cancer Etiology, Department of Population Science, Beckman Research Institute, City of Hope, California, United States of America; 30 Division of Epidemiology, Department of Medicine, Vanderbilt Epidemiology Center, Nashville, Tennessee, United States of America; 31 Vanderbilt-Ingram Cancer Center, Vanderbilt University School of Medicine, Nashville, Tennessee, United States of America; 32 Sylvester Comprehensive Cancer Center and Department of Epidemiology and Public Health, University of Miami Miller School of Medicine, Miami, Florida, United States of America; 33 Epidemiology and Biostatistics Program, Division of Cancer Epidemiology and Genetics, National Cancer Institute, Bethesda, Maryland, United States of America; 34 The Cancer Institute of New Jersey, Robert Wood Johnson Medical School, New Brunswick, New Jersey, United States of America; 35 Division of Cancer Epidemiology and Genetics, National Cancer Institute, Bethesda, Maryland, United States of America; 36 Institute for Medicine and Public Health, Vanderbilt Epidemiology Center, Nashville, Tennessee, United States of America; 37 Divisions of Genetics and Endocrinology and Program in Genomics, Children’s Hospital Boston, Boston, Massachusetts, United States of America; 38 Department of Statistics, University of Oxford, Oxford, United Kingdom; 39 V. A. Medical Center, Jackson, Mississippi, United States of America; University of California San Diego and The Scripps Research Institute, United States of America

## Abstract

While genome-wide association studies (GWAS) have primarily examined populations of European ancestry, more recent studies often involve additional populations, including admixed populations such as African Americans and Latinos. In admixed populations, linkage disequilibrium (LD) exists both at a fine scale in ancestral populations and at a coarse scale (admixture-LD) due to chromosomal segments of distinct ancestry. Disease association statistics in admixed populations have previously considered SNP association (LD mapping) or admixture association (mapping by admixture-LD), but not both. Here, we introduce a new statistical framework for combining SNP and admixture association in case-control studies, as well as methods for local ancestry-aware imputation. We illustrate the gain in statistical power achieved by these methods by analyzing data of 6,209 unrelated African Americans from the CARe project genotyped on the Affymetrix 6.0 chip, in conjunction with both simulated and real phenotypes, as well as by analyzing the FGFR2 locus using breast cancer GWAS data from 5,761 African-American women. We show that, at typed SNPs, our method yields an 8% increase in statistical power for finding disease risk loci compared to the power achieved by standard methods in case-control studies. At imputed SNPs, we observe an 11% increase in statistical power for mapping disease loci when our local ancestry-aware imputation framework and the new scoring statistic are jointly employed. Finally, we show that our method increases statistical power in regions harboring the causal SNP in the case when the causal SNP is untyped and cannot be imputed. Our methods and our publicly available software are broadly applicable to GWAS in admixed populations.

## Author Summary

This paper presents improved methodologies for the analysis of genome-wide association studies in admixed populations, which are populations that came about by the mixing of two or more distant continental populations over a few hundred years (e.g., African Americans or Latinos). Studies of admixed populations offer the promise of capturing additional genetic diversity compared to studies over homogeneous populations such as Europeans. In admixed populations, correlation between genetic variants exists both at a fine scale in the ancestral populations and at a coarse scale due to chromosomal segments of distinct ancestry. Disease association statistics in admixed populations have previously considered either one or the other type of correlation, but not both. In this work we develop novel statistical methods that account for both types of genetic correlation, and we show that the combined approach attains greater statistical power than that achieved by applying either approach separately. We provide analysis of simulated and real data from major studies performed in African-American men and women to show the improvement obtained by our methods over the standard methods for analyzing association studies in admixed populations.

## Introduction

Genome-wide association studies (GWAS) are the currently prevailing approach for identifying genetic variants with a modest effect on the risk of common disease, and have identified hundreds of common risk variants for a wide range of diseases and phenotypes [1], [2]. Although GWAS have initially focused on populations of European ancestry, studies of other populations will capture additional genetic diversity that may be absent or present only at low frequency in Europeans. GWAS in non-Europeans will often involve admixed populations, such as African Americans and Latinos, with recent ancestry from two or more ancestral populations [3], [4].

GWAS disease mapping in homogeneous populations relies on linkage disequilibrium (LD) between nearby markers to identify SNP association [5]. Admixed populations exhibit another form of LD at a coarse scale (admixture-LD) due to chromosomal segments of distinct ancestry [6]. This enables admixture mapping (mapping by admixture-LD) to be an effective approach for identifying disease genes in admixed populations [7]–[14]. As genotyping costs have decreased, however, GWAS have become an increasingly appealing alternative. Although GWAS and admixture mapping have historically been viewed as distinct approaches, GWAS in admixed populations can in theory capture both SNP and admixture association signals, which have been shown to contain independent information [15]. To date, GWAS in such populations have either considered SNP association only [3], [16], [17], or SNP and admixture association separately [4]. We show below that combining these signals leads to increased statistical power because case-only admixture association statistics contain information independent from case-control SNP association statistics.

It is important to complement theoretical methods development with empirical evaluation on large real data sets. To this end, we have evaluated our methods using 6,209 unrelated African Americans from the CARe cardiovascular consortium as well as 5761 unrelated African-American women from a GWAS for breast cancer. We ran comprehensive simulations based on real genotypes and phenotypes simulated under a variety of assumptions. Our main focus was on case-control phenotypes, in which case-only admixture association is particularly valuable. Our analysis of simulated and real (coronary heart disease, type 2 diabetes and breast cancer) case-control phenotypes shows that our combined SNP and admixture association approach attains significantly greater statistical power than can be achieved by applying either approach separately. Although our main focus is on case-control phenotypes, we also provide a detailed evaluation of association statistics for quantitative phenotypes, using simulated and real (LDL and HDL cholesterol) phenotypes.

Since the general assumption in GWAS is that the causal SNP is not directly typed in the study, it is important to assess how the newly introduced scores perform in the context of genotype imputation. First, we show that imputation accuracy is marginally improved when local ancestry is taken into account in the imputation procedure. Second, our analysis in African Americans shows that for case-control studies our methods for combining SNP and admixture association outperform other approaches even in the presence of imputation. Finally, we show that when the causal SNP is not typed and cannot be reliably imputed our methods yield higher statistical power at finding the region harboring the causal variant when compared to previous approaches. Based on these findings we provide recommendations for the use of our combined approach in GWAS of admixed populations.

## Results

### CARe data set

We analyzed data from 6,209 unrelated African Americans from the CARe consortium who were genotyped on the Affymetrix 6.0 chip, and merged in genotype data from the HapMap3 project (see Methods) [18]. We ran principal components analysis (PCA) on the merged data using the EIGENSOFT software, using only the CEU, YRI and CHB populations from HapMap3 to compute principal components [19], [20]. The CARe samples generally occupy intermediate positions between CEU and YRI, consistent with previous work (Figure S1) [21], [22]. We ran the HAPMIX program for inferring local ancestry (0, 1 or 2 European chromosomes) at each location in the genome on the CARe samples, using phased CEU and YRI haplotypes from HapMap3 as reference [23]. HAPMIX was run in a mode that assigns European or African ancestry to each allele, thus resolving the local ancestry of each allele when both genotype and local ancestry were heterozygous (see Methods). We defined genome-wide ancestry for each sample as the average of local ancestry estimates across the genome (scaled to 0.0, 0.5 or 1.0). Genome-wide European ancestry estimates had a mean of 19.2% and standard deviation of 12.0% across samples (consistent with previous work [21], [22]), and were >99% correlated with the top eigenvector from PCA analysis. We defined average local ancestry at each location in the genome as the average of local ancestry values across samples. A plot of average local ancestry shows no unusual peaks in average local ancestry (Figure S2), consistent with the fact that the full set of CARe samples were not ascertained for a specific disease phenotype and thus would not be expected to produce an admixture peak, and confirming that HAPMIX does not produce artifactual deviations in average local ancestry. Importantly, we note that local ancestry can be estimated using any of the local ancestry inference methods that have been proposed (e.g. [7], [23], [24]), as long as they are accurate and do not produce artifactual deviations in average local ancestry. We mention in passing that very strong selection since admixture for an allele differing in frequency between Europeans and West Africans could in theory produce a true local ancestry deviation, and our data could be used to provide an upper bound on the size of any such effect. We do not pursue this here.

### Overview of association statistics for case-control phenotypes

We used the Armitage trend test with correction for genome-wide ancestry as a baseline for the evaluation of other approaches, as this approach was used in previous association analyses using CARe data [25] (see Methods). Next, we considered a SNP association score conditioned on local ancestry, as well as a case-only admixture score which evaluates the causal hypothesis that, restricting to disease cases, the proportion of European ancestry at the candidate locus differs from the genome-wide proportion [7] (see Methods). Historically, an advantage of admixture association was that disease mapping could be performed using a coarse set of markers, due to the large size of ancestry segments and the resulting admixture linkage disequilibrium [22]. However, even when GWAS data are available, admixture scores that compare disease cases to the same disease cases elsewhere in the genome contain different information than SNP association scores that compare cases to controls; the additional information is particularly valuable when the causal SNP has very different allele frequencies in the ancestral populations. One possibility is to add the SNP association score conditioned on local ancestry to the admixture score to produce a χ^2^(2dof) score, but as we show below, the higher degrees of freedom leads to a reduction in statistical power. We instead propose a mixed χ^2^(1dof) score that jointly evaluates both SNP and admixture association using a single SNP odds ratio, by using the implied ancestry odds ratio (see Methods). An important question is whether the odds ratio conditioned on African local ancestry differs from the odds ratio conditioned on European local ancestry, as this has implications for fine-mapping the causal SNP. This can be addressed by comparing the χ^2^(1dof) SNP association score conditioned on local ancestry to a χ^2^(2dof) SNP association score which allows different odds ratios for African versus European local ancestry (see Methods). A final question, important in the context of localizing the causal SNP, is whether the ancestry odds ratio is fully explained by the SNP odds ratio. This can be addressed by comparing the χ^2^(1dof) MIX score that accounts for both admixture and case-control signal using a single SNP odds ratio and the χ^2^(2dof) SUM score that allows for independent SNP and ancestry odds ratios.

We also explored whether it is necessary to assign African or European ancestry to each allele for a sample and SNP in which both local ancestry and genotype are heterozygous. Although the HAPMIX algorithm supports this functionality, it represents a significant complexity, particularly if representing local ancestry inference in terms of real-valued probabilities. We focus below on scores based on diploid local ancestry (AA, AE or EE) that do not require this extra information, and show that these scores perform nearly as well as scores that are based on haploid local ancestry (A or E) for each of two chromosomes with local ancestry inference and phasing performed jointly.

### Simulations of case-control phenotypes

We randomly selected 100,000 autosomal SNPs and, for each SNP, assigned simulated phenotypes based on either a null model or causal model for that SNP. Under the null model, we chose 1,000 cases and 1,000 controls at random. Under the causal model, we chose 1,000 cases and 1,000 controls corresponding to odds ratios *R* = 1.2,1.5 or 2.0 (see Methods). Thus, our simulations use real genotypes, with simulated phenotypes that are different for each SNP being tested (and different for each value of *R*). This framework automatically leads to admixture association signals as would exist with real phenotypes: for example, a causal SNP in which the risk allele has higher frequency in Europeans than in Africans will lead to the selection of 1,000 cases with higher than average European ancestry at the disease locus.

We compared 5 scores: Armitage trend test with correction for genome-wide ancestry (ATT), SNP association conditioned on local ancestry (SNP1), admixture association using cases only (ADM), sum of SNP1 and ADM (SUM), and our new mixed score (MIX). All of these are χ^2^(1dof) scores, except for SUM which is χ^2^(2dof). We note that the strength of the induced admixture signal at highly differentiated SNPs (as measured by the ancestry odds ratio) in the simulated data fits the model assumed in the MIX score.

**Figure 1:**
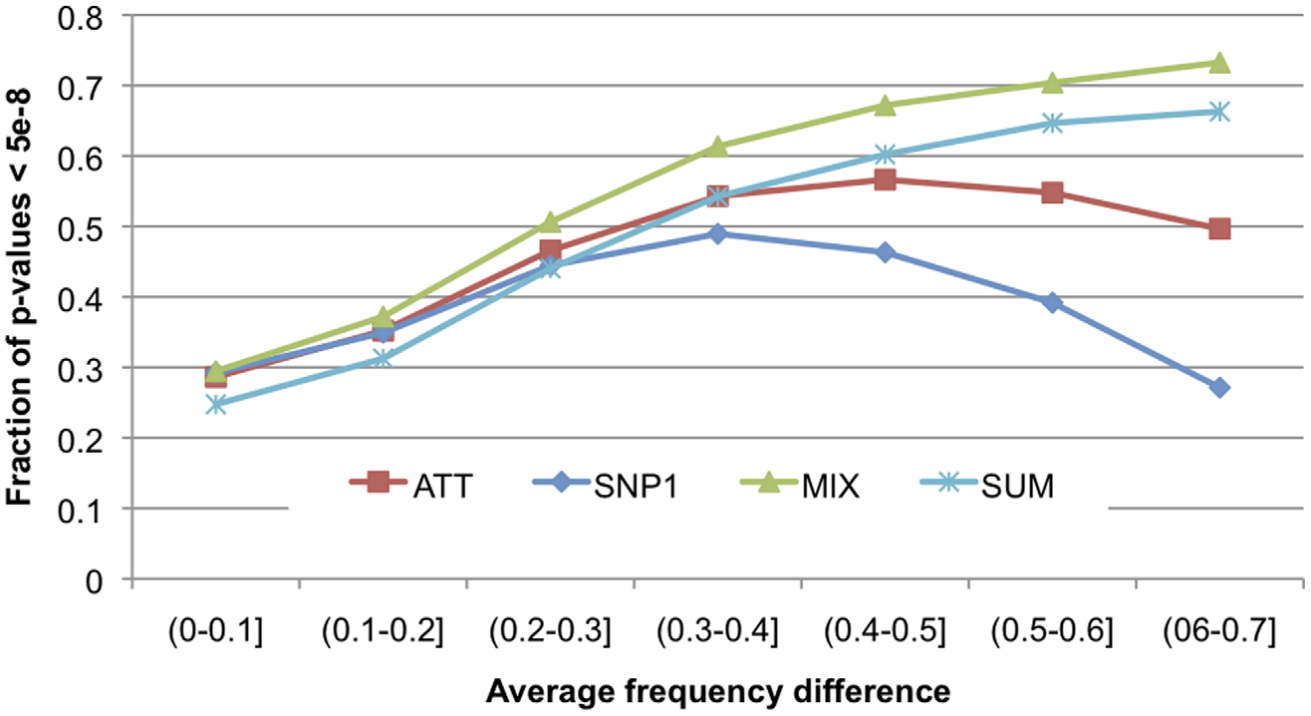
Statistical power of SNP1, ATT, MIX scores as a function of population differentiation. We plot the average power of each score as a function of allele frequency difference between CEU and YRI, for the R = 1.5 simulation only.

In Table 1 (Typed Genotypes) we display results obtained by all scores averaged across all SNPs, and averaged across SNPs with CEU versus YRI allele frequency difference of at least 0.4, roughly the top decile of differentiation. We used a p-value cutoff of 5e-08 for all scores except ADM for which a threshold of 1e-05 was employed. The different ADM threshold is motivated by the smaller number of independent hypotheses tested across the genome in an admixture scan (an effect of the large size of the ancestry segments) [6], [7]. The MIX score attains 8% higher power than the ATT score for random SNPs (24% higher power for SNPs in the top decile) at *R* = 1.5. The SNP1 score, which is conditioned on local ancestry, is analogous to disease mapping in Europeans or Africans (see Text S1). Thus, disease mapping in African Americans using the MIX achieves an increase in statistical power of 13% for random SNPs and of 67% for SNPs in the top decile of population differentiation over disease mapping in Europeans or Africans. This advantage is obtained both because MIX is a more powerful score than ATT, and because of the inherent advantage of disease mapping in admixed populations, which contain more polymorphic variation. As expected, the advantage of the MIX score is greatest for SNPs with large allele frequency differences between Africans and Europeans, for which admixture association produces a strong signal (Table 1 (Typed Genotypes) and Figure 1). We obtained similar results for a variant of the MIX score based on haploid local ancestry with joint local ancestry inference and phasing (Text S1). Thus, fully powered association statistics in admixed populations do not require joint local ancestry inference and phasing. We finally note that the heterogeneity score that tests for differences in effect size for African versus European local ancestry (HET) attained average values between 0.99–1.01 (data not shown), exactly as expected since simulated phenotypes did not involve heterogeneity in effect size.

**Table 1:** Average statistical power of simulated case-control scores in African Americans computed using (a) typed or (b) imputed genotypes.

Typed Genotypes						
	R = 1.2 random	R = 1.2 Δ>0.4	R = 1.5 random	R = 1.5 Δ>0.4	R = 2.0 random	R = 2.0 Δ>0.4
ATT χ2(1dof)	0.0017	0.0026	0.3803	0.5533	0.8351	0.9769
SNP1 χ2(1dof)	0.0014	0.0012	0.3628	0.4181	0.8279	0.9362
ADM χ2(1dof)	0.0001	0.0013	0.0081	0.0903	0.0737	0.6306
SUM χ2(2dof)	0.0012	0.0028	0.3555	0.624	0.8287	0.9874
MIX χ2(1dof)	0.0021	0.0046	0.4131	0.6899	0.8486	0.9907

Imputed Genotypes						
	R = 1.2 random	R = 1.2 Δ>0.4	R = 1.5 random	R = 1.5 Δ>0.4	R = 2.0 random	R = 2.0 Δ>0.4
ATT χ2(1dof)	0.0010	0.0008	0.2871	0.2988	0.7620	0.7762
ATT-dose χ2(1dof)	0.0010	0.0008	0.3009	0.3134	0.7775	0.7938
SNP1 χ2(1dof)	0.0009	0.0007	0.2673	0.3013	0.7483	0.8748
ADM χ2(1dof)	0.0001	0.0013	0.0081	0.0903	0.0737	0.6306
SUM χ2(2dof)	0.0007	0.002	0.2668	0.5086	0.7567	0.9729
MIX χ2(1dof)	0.0013	0.0034	0.3184	0.5915	0.778	0.9786

For each score we list the proportion of SNPs for which the score attains genome-wide significance (defined as P<5e-08 for all scores except ADM, P<1e-05 for ADM), for random SNPs as well as SNPs in the top decile of population differences (Δ>0.4), for *R* = 1.2, *R* = 1.5, *R* = 2.0 simulations (see main text). For R = 1.0 the power is 0 for all scores. In general the MIX score shows an increase in statistical power relative to the ATT score, and a further increase in power relative to the SNP1 score, which is analogous to disease mapping in European or African populations. ATT-dose denotes ATT test using imputation dosages.

We also assessed all scores at null simulated data (R = 1) using the standard genomic control [26] statistic λ_GC_ which attained a value of 1.001 for MIX, 0.986 for SNP1 and 0.999 for the ATT score, respectively. We observed a λ_GC_ of 1.101 for the ADM score, which is suggestive of inflation, although we note that, for 1000 cases and a thousand independent genomic regions (as expected in the ADM score), a λ_GC_ of 1.101 can arise by chance. However, since multiple factors (e.g. deviations from random mating, correlation in errors of local ancestry estimates) could potentially lead to inflation of the ADM statistic, we have also devised an admixture statistic, ADMGC that incorporates the empirical variance of the average local ancestry (see Methods). It can be shown that ADMGC is equivalent to dividing the ADM statistics by λ_GC_. Furthermore, we show how to incorporate ADMGC within the MIX framework to obtain a new version of our score (MIXGC) that incorporates the new admixture component. As expected, both ADMGC and MIXGC attain λ_GC_ of 1.000 (data not shown) in simulated null data. We note that MIXGC should be used when there is significant indication of inflation. As this was not the case here, we chose to use MIX for all results below.

We also assessed the performance of our scores when the disease model assumptions are not met. We simulated causal SNPs under various disease models such as dominant and recessive or when two causal independent SNPs are present within an admixture block. To simulate two causal independent SNPs within same admixture block, we restricted to SNPs less than 5Mb apart and with LD less than .1 (as measured by r^2^). Results in Table S3 confirm that for most scenarios studied the MIX score outperforms the standard ATT score with correction for genome-wide ancestry. Interestingly, when restricting to 2 causal SNP scenario in which one of the causal is in the top decile of differentiation (which induces a strong admixture signal) we observe that the SUM score outperforms all other scores in terms of power, showing the potential utility of this score at loci with multiple causal variants.

We also looked at heterogeneous effects across Europeans and Africans by simulating 100,000 causal SNPs with R = 1.5 (under no heterogeneity) and assessing the scores at SNPs with different levels of LD with the simulated causal in the two populations. Different LD across populations will induce heterogeneous effects as a function of the allele frequencies and the population specific LD pattern. Results in Figure S4 show that under small heterogeneous effects (difference in observed odds ratios <0.25), the MIX score outperforms the other scores in terms of power while in the presence of larger heterogeneity all scores are underpowered in this simulation.

### Genotype imputation in African Americans

Due to the limited number of markers present on the genotyping platforms, it is often the case that the causal SNP is not directly typed within the GWAS. However, genotypes typed in a study can be used as predictors, in conjunction with haplotypes over denser sets of SNPs from external repositories of human variation such as the HapMap [27], to impute genotypes at SNPs untyped in the current study. Genotype imputation has been widely used as a method for boosting statistical power in association and fine-mapping studies as well as in meta-analysis that combines information across studies as a tool for increasing the number of markers interrogated for association with the phenotype [28]–[30]. Multiple methods [31], [32] have been proposed for solving the imputation problem and have been shown to be very accurate when the haplotypes used as a reference panel provide a good match to the study population [28], [30], [33]. In admixed populations various imputation approaches have been proposed ranging from assigning global weights to the reference panels based on empirical estimates of ancestry [30], to assigning coalescent-based weights to each of the reference haplotypes in every sample and every locus in the genome [34]. A standard approach for imputation in African Americans is to use a reference panel composed of European and African chromosomes [18], [25]. Recent work has shown that imputation conditional on local ancestry estimates can boost the overall accuracy when compared to imputation based cosmopolitan reference panels that contain haplotypes from all the ancestral populations [24], [35]. Here, through the use of real CARe genotypes, we show that imputation conditional on local ancestry yields a small improvement in imputation accuracy in African Americans. Our local ancestry aware imputation framework uses, at every locus in the genome, a reference a panel of haplotypes that is specified by the local ancestry (see Methods).

**Figure 2:**
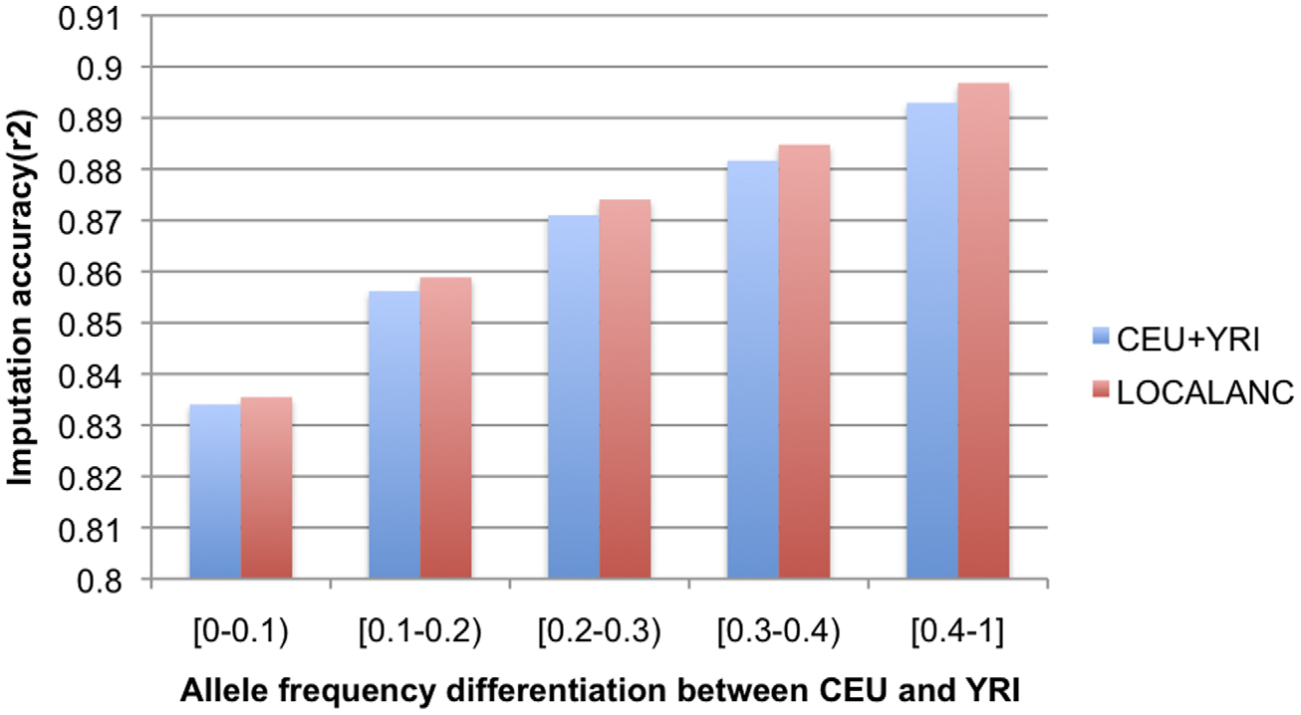
Imputation accuracy as a function of population differentiation. We plot the average imputation accuracy as a function of allele frequency difference between CEU and YRI both when CEU+YRI was used as reference and when using the local ancestry aware framework.

Following a standard masking approach, we masked 100,000 SNPs at random from the CARe data, imputed them and assessed imputation accuracy using a standard accuracy measure, the squared correlation between imputed and true ‘masked’ genotypes. We observe an average imputation r^2^ of 0.858 when our local ancestry aware framework is used, as opposed to 0.855 under the standard cosmopolitan approach, confirming that there is a small gain in accuracy by conditioning imputation on local ancestry. We observe a smaller improvement in imputation performance than the one reported in [24], [35] which can be an effect of different imputation methods as well as of difference in size of reference HapMap panels used. We employed a much larger set of reference haplotypes (HapMap phase 3 versus phase 2) in imputation that could potentially reduce the effect of incorporating local ancestry. Importantly, we note that the gain in accuracy is observed across all SNPs and leads to a small gain in statistical power for association (see Figure 2 and Table S1). We also point out that a large percentage of the imputed SNPs show a large difference in imputation performances between the European and African segments (see Figure S3). Roughly 40% of the imputed SNPs show accuracies differing by at least 0.1 in terms of squared correlation in European versus African segments with 26% being more accurately imputed in European segments versus 14% in African segments.

### Case-control association statistics at imputed SNPs

A straightforward approach for extending association statistics at imputed SNPs is to use the maximum likelihood estimates for unobserved genotypes. Although this procedure does not fully account for the uncertainty in the imputed genotypes, it has been previously shown to perform well when there is considerable confidence in the imputed genotype calls. Throughout this paper we compute statistics over the maximum likelihood genotype calls. Although our novel scores could potentially be improved by fully incorporating the imputation uncertainty in the likelihood framework we note that the MIX score outperforms the standard ATT score, even when the ATT score accounts for the imputation uncertainty through the use of dosages instead of maximum likelihood genotype calls (see Table 1 (Imputed Genotypes)). An important aspect of applying the case-control statistics to imputed data in African Americans is to properly account for the difference in imputation quality between African and European segments. We accomplish this by adjusting the observed allelic odds ratio as a function of imputation quality in the MIX and SNP1 score (see Text S1).

We masked the 100,000 SNPs that were used for simulation of phenotypes and imputed genotypes at these SNPs using our local ancestry aware imputation framework (see Methods). We computed the scores over the imputed genotype calls with the results displayed in Table 1 (Imputed Genotypes). As expected, scores over imputed data show a reduction in statistical power because of the noise introduced by imputation errors. Importantly, we note that, similarly to typed data, the MIX score outperforms the other scores in terms of power, attaining 11% higher statistical power than the ATT score for random imputed SNPs (97% higher power for imputed SNPs in the top decile of allele frequency differentiation) at *R* = 1.5. Even when the ATT score allows for imputation uncertainty in the form of dosages, there is still a gain in statistical power of 6% at random SNPS (R = 1.5) of MIX over ATT. We also note that adjusting the MIX score for different imputation qualities leads to a small improvement in statistical power at imputed SNPs (see Table S1).

### Scoring when the causal SNP is not typed and cannot be imputed

An important aspect in disease scoring statistics is to assess their performance when the causal SNP is untyped and, due to various reasons (e.g. not present in the reference panel), cannot be imputed. To address this scenario we randomly picked 100,000 autosomal SNPs and simulated case-control phenotypes for R = 1.5 using the methodology described above. For all the SNPs we evaluated the statistics at 40 SNPs in the neighborhood of the simulated SNP and, for each score, computed the maximum statistic in this region by either masking the simulated causal SNP or by including it in the computation of the maximum. Results in Table 2 show that, both when the causal SNP is present in the data and when it is absent from the data, the MIX score outperforms all the other scores in terms of power.

**Table 2:** Disease scoring when the causal SNP is not typed or imputed.

Score	Average maximum χ^2^ value		Proportion of regions that are genome wide significant	
ATT χ2(1dof)	26.17	**18.08**	0.3834	**0.1752**
SNP1 χ2(1dof)	25.47	**17.52**	0.3622	**0.1618**
ADM χ2(1dof)	4.23	**4.22**	0.0135	**0.0134**
SUM χ2(2dof)	28.62	**20.69**	0.3571	**0.1675**
MIX χ2(1dof)	27.46	**19.05**	0.4158	**0.1988**

We list the average maximum statistic and the percentage of times it attains genome wide significance (defined as P<5e-08 for all scores except ADM, P<1e-05 for ADM) for each of the case-control scores obtained in a region of 40 SNPs centered around the 100,000 simulated causal SNPs with R = 1.5. The results obtained when the score at the simulated causal SNP was removed from the computation of the maximum are denoted in bold. The MIX score outperforms the other scores both when the causal is present or unobserved in the data.

### Application to real phenotypes

#### Application to coronary heart disease and type 2 diabetes case-control phenotypes

As a sanity check we evaluated these scores using data from the CARe study for two case-control phenotypes: coronary heart disease (CHD) and type 2 diabetes (T2D), for which associations at several loci have been reported previously [25]. Results for genotyped and imputed SNPs are displayed in Table 3 (see Methods). Because CARe is a cohort study, the number of cases for CHD or T2D is much smaller than the number of controls, so that in addition to being generally underpowered, in this analysis the potential advantage of incorporating case-only admixture information is marginal (see Table S2). Indeed, as expected, the ATT and MIX scores generally produce similar results, though in some instances the ATT score slightly outperforms the MIX score, and in this example the MIX score was not the “Best score” at any of the five loci. Interestingly, we observe that two of the top SNPs (rs1333047 and rs6475606) show a relatively large HET score (HET = 6.84, P-value = 0.009 for rs1333047 and HET = 6.79 P-value = 0.009 for rs6475606), implying different odds ratios conditional on African versus European local ancestry. We believe that these SNPs may tag unobserved causal SNP(s) with very different LD patterns in Africans versus Europeans. However, we cannot rule out the alternate explanation that the causal SNP has heterogeneous effect size (for example, due to gene-gene interaction with another causal SNP in the same region that has different allele frequencies in Africans and Europeans).

**Table 3:** Results for CHD and T2D case-control phenotypes.

CHD										
SNP	chrom	position (build35)	CEUfreq	YRIfreq	ATT	SNP1	ADM	SUM	HET	MIX
rs17577085	5	141,843,788	0.11	0.00	2.66	1.54	1.46	2.00	0.00	2.06
rs4244029*	5	141,893,025	0.08	0.27	2.66	2.84	1.31	3.06	0.56	2.51
Best score	5	-	-	-	2.66	2.84	1.93	**3.06**	-	2.51
rs325105	6	147,805,960	0.47	0.012	2.62	1.65	0.81	1.57	0.65	2.15
rs325129*	6	147,848,836	0.25	0.74	3.22	2.55	1.05	2.57	0.26	3.12
Best score	6	-	-	-	**3.22**	2.86	1.18	2.79	-	3.13
rs6475606	9	22,071,850	0.5	0.01	1.87	2.72	0.11	2.11	2.04	2.38
rs1333047*	9	22,114,504	0.49	0.99	2.32	3.64	0.00	2.95	2.05	2.96
Best score	9	-	-	-	2.50	**3.64**	0.32	2.95	-	2.99

T2D										
SNP	chrom	position (build35)	CEUfreq	YRIfreq	ATT	SNP1	ADM	SUM	HET	MIX
rs13424957	2	165,575,897	0	0.28	4.58	4.19	0.61	3.76	0.00	4.46
rs13396952*	2	165,562,786	0.02	0.3	4.41	4.04	0.57	3.61	0.13	4.29
Best score	2	-	-	-	**4.58**	4.19	1.01	3.76	-	4.46
rs7901695	10	114,744,078	0.28	0.53	4.11	4.36	0.75	4.01	0.16	3.97
rs7903146*	10	114,748,339	0.25	0.29	5.37	5.03	0.80	4.69	0.19	5.05
Best score	10	-	-	-	**5.37**	5.03	1.25	4.69	-	5.05

For each CHD region, we list results for each score (-log in base 10 of the p-value) for the originally implicated genotyped SNP, the imputed (* denotes imputed SNPs) or genotyped SNP producing the most significant P-value in the region and the best score for each of the five scores. Analogous to CHD, for each T2D region. The value achieving the smallest p-value is denoted in bold.

Finally, we note that due to the fundamental difference between the asymptotically equivalent goodness-of-fit (ATT) and likelihood-ratio χ^2^(1dof) tests (MIX), the scores may differ in either direction, but the likelihood-ratio approach used in the MIX score is theoretically appropriate (see Text S1).

#### Application to *FGFR2*, a known locus associated with breast cancer

For a test analysis with a larger number of cases and potentially greater case-only admixture information, we also evaluated the above scores at the known FGFR2 breast cancer locus [36] in 3,153 African American cases and 2,831 controls from a GWAS for breast cancer. We focused our analysis on this locus because it has been extensively fine-mapped in African American populations [37] with the strongest reported association at SNP rs2981578. We performed imputation in this region and applied our scores to all SNPs within 1MB of this SNP. As expected the highest signals of association were observed at SNP rs2981578 (see Table 4). We note that both the SUM and MIX scores outperform the ATT test showing the utility of incorporating case only admixture association in the scoring statistics, especially in the presence of strong admixture signals. Since the SUM likelihood generalizes the MIX likelihood by allowing for an extra free parameter (the ancestry odds ratio), the difference in the SUM-MIX can be viewed as a test of whether the ancestry odds ratio inferred from the SNP allelic odds ratio R fits the observed ancestry odds ratio in the data. Multiple causal variants within the same admixture block could potentially create a large admixture signal that is not captured by the odds ratio at each of the causal SNPs (see Table S4). Thus, the difference between the SUM = ADM+SNP1 score (χ^2^(2 dof)  = 22.74) and the MIX score (χ^2^(1 dof)  = 17.04) provides some evidence (χ^2^(1 dof)  = 5.7, P-value = 0.016) that rs2981578 may not be the unique causal variant at the FGFR2 locus. We also note that the HET score (χ^2^(1 dof)  = 1.80) provides little to no evidence in support of the hypothesis of heterogeneity at this SNP. Complete results of the breast cancer GWAS will be presented elsewhere (C. Haiman and colleagues, unpublished data).

**Table 4:** Results obtained at FGFR2 locus, SNP rs2981578 using MACH imputation.

	ATT	ADM	MIX	SNP1	HET	SUM
χ^2^ value	13.99	6.16	17.04	16.57	1.80	22.74
-log10(p-value)	3.74	1.88	4.44	4.33	0.75	4.94

We list the χ^2^ values along with the –log(p-value) obtained by the case-control scoring statistics showing that incorporating the admixture signal yields increased results over the standard ATT test with correction for global ancestry. We note that SNP rs2981578 shows the highest scores in the region.

### Overview of association statistics for continuous phenotypes

We again used the Armitage trend test with correction for genome-wide ancestry as the baseline for our analyses. We also considered a SNP association score conditioned on local ancestry, as well as an admixture score that associates the local ancestry to the continuous phenotype with genome-wide ancestry as a covariate. (There is no analogue to a case-only admixture score for quantitative traits). As in the dichotomous case, we summed the SNP association score conditioned on local ancestry with the admixture score to produce a χ^2^(2dof) score, but show below that the higher degrees of freedom lead to a reduction in statistical power. Finally, we considered a χ^2^(1dof) heterogeneity score that tests for a difference in effect size conditional on African or European ancestry, by comparing a model that allows different effect sizes to a model with a uniform effect size (see Methods).

### Simulations of quantitative phenotypes

Analogous to simulations of dichotomous phenotypes, for 100,000 randomly chosen SNPs we used CARe genotypes and simulated phenotypes for 2,000 samples based on a null model or a causal model with effect sizes *ε* = 0.05,0.10,0.20 (see Methods).

We compared 4 scores: Armitage trend test with correction for genome-wide ancestry (QATT), SNP association conditioned on local ancestry (QSNP1), local ancestry admixture association (QADM), and sum of QSNP1 and QADM (QSUM). All of these are χ^2^(1dof) scores, except for QSUM which is χ^2^(2dof). Results are displayed in Table 5 (Typed Genotypes). We display results averaged across all SNPs, and averaged across SNPs with CEU versus YRI allele frequency difference of at least 0.4, roughly the top decile of differentiation. We see that the Armitage trend test (QATT) outperforms the other scores. Here, there is no advantage to incorporating admixture scores, since no case-only score is available and since summing SNP and admixture association scores (QSUM) loses statistical power due to increased degrees of freedom. We finally note that the heterogeneity score that tests for differences in effect size for African versus European local ancestry (QHET) attained average values between 0.99–1.01 (data not shown), exactly as expected since simulated phenotypes did not involve heterogeneity in effect size. As in the case of the dichotomous phenotypes, we masked the 100,000 SNPs followed by imputation and we applied the above scores on the imputed genotypes (see Table 5 (Imputed Genotypes)). Although the overall statistical power decreases for all scores because of imputation errors, we note that as before, QATT outperforms the other scores in terms of statistical power.

**Table 5:** Average statistical power of simulated quantitative scores in African Americans.

Typed Genotypes						
	*ε* = 0.05random	*ε* = 0.05 Δ>0.4	*ε* = 0.10 random	*ε* = 0.10 Δ>0.4	*ε* = 0.20 random	*ε* = 0.20 Δ>0.4
QATT χ^2^(1dof)	0.0013	0.0009	0.2165	0.3223	0.8566	0.9883
QSNP1 χ^2^(1dof)	0.0012	0.0005	0.1951	0.2087	0.8437	0.9422
QADM χ^2^(1dof)	0	0.0001	0.0004	0.0048	0.0229	0.2594
QSUM χ^2^(2dof)	0.0006	0.0003	0.1636	0.2473	0.8353	0.9839

Imputed Genotypes						
	*ε* = 0.05random	*ε* = 0.05 Δ>0.4	*ε* = 0.10 random	*ε* = 0.10 Δ>0.4	*ε* = 0.20 random	*ε* = 0.20 Δ>0.4
QATT χ^2^(1dof)	0.0008	0.0009	0.1526	0.1677	0.7853	0.7993
QSNP1 χ^2^(1dof)	0.0007	0.0008	0.1346	0.1398	0.7663	0.7772
QADM χ^2^(1dof)	0	0.0001	0.0004	0.0048	0.0229	0.2594
QSUM χ^2^(2dof)	0.0004	0.0004	0.1115	0.1245	0.7617	0.7762

For each score we list the proportion of SNPs for which the score attains genome-wide significance (defined as P<5e-08 for all scores except QADM, P<1e-05 for QADM), for random SNPs as well as SNPs in the top decile of population differences (Δ>0.4), 0, *ε* = 0.05, *ε* = 0.10, *ε* = 0.20 simulations (see main text). For *ε* = 0, the power is 0 for all scores. Imputed Genotypes: The same 100,000 SNPs were masked, followed by imputation, and the imputed genotypes were scored and presented as in Typed Genotypes.

### Application to real quantitative phenotypes

We evaluated the above scores using data from two quantitative phenotypes from CARe, LDL and HDL cholesterol, for which associations at several loci have previously been reported. Results for genotyped and imputed SNPs in the region are displayed in Table S4. As in our simulations, the QATT score yields the best performance the majority of the time. However, one aspect of the results is of particular interest. Multiple LDL and HDL SNPs on chromosome 2 produce strong admixture association (QADM) scores, with the result that the χ^2^(2 dof) QSUM score outperforms the χ^2^(1 dof) ATT score. We point out that the presence of multiple causal variants, or alternatively an untyped/unimputed variant with large allele frequency differentiation, may invalidate the assumptions made by the QATT score and lead to poor performance. This suggests that the QSUM score can be of value in a minority of instances where strong admixture associations exist. We caution that in such cases an additional multiple hypothesis testing correction may be needed and that the QSNP1 score conditioned on local ancestry will be needed for localization [38].

## Discussion

Incorporating admixture association signals into GWAS of admixed populations is likely to be particularly informative for diseases for which risk differs depending on ancestry. Cardiovascular disease (CVD) is a prime example, as African ancestry is associated to higher CVD mortality and to CVD risk factors such as hypertension, serum lipid levels and left ventricular hypertrophy [39]–[41]. Other diseases for which African ancestry is a risk factor include prostate cancer, diabetic retinopathy, lupus and uterine fibroids [42]–[45]. Although we have focused here on African Americans, our methods are broadly applicable to other admixed populations.

By analyzing real and simulated case-control phenotypes, we have shown that the MIX score, which incorporates both SNP and admixture association signals, yields a significant increase in statistical power over commonly used scores such as the Armitage trend test with correction for global ancestry. For randomly ascertained quantitative traits, in contrast to case-control phenotypes there is no case-only admixture score and thus no benefit from joint modeling of admixture and SNP association. Thus, for quantitative phenotypes, in general, the QATT score yields higher statistical power than other compared scores. Therefore, we recommend the use of MIX and QATT scores for dichotomous and quantitative traits, respectively, in future GWAS in admixed populations. However, we note that in various scenarios (e.g., multiple causal variants, heterogeneous effects, absence of the causal variant from the typed or imputed markers) assumptions made by the MIX and QATT may be invalid and using them can lead to poor performance. To this extent, we recommend that special consideration be given to regions with high signals of admixture association, in which the SUM and QSUM scores may produce higher association signals than MIX and QATT. As a future direction, we note that an improved score for non-randomly ascertained quantitative traits could potentially be developed, which would generalize both the MIX score for dichotomous traits and the QATT score for randomly ascertained quantitative traits.

As GWAS in European populations have demonstrated, association statistics need not be limited to SNPs that have been genotyped, because imputation algorithms that we and others have developed can be used to infer the genotypes of untyped SNPs by making use of haplotype information from HapMap. Our methods also perform well in the setting of imputation, when the causal SNP is not genotyped. As future work we consider the extension of our likelihood based scores to fully account for imputation uncertainty, where a promising direction is to define the likelihood as a full integration over the missing data given the observed data and the parameters of the model [46], [47].

Our results using simulated phenotypes show that, although benefiting from a reduced multiple-hypothesis testing burden, the admixture scoring yields lower power for finding associations when compared to SNP association scoring. An explanation is the limited number of SNPs that show high allelic differentiation among the ancestral populations (e.g., in our simulations only 7.6% of the SNPs have an allelic differentiation greater than 0.4 between Europeans and Africans). However, we note that the question of whether there exists a combined SNP and admixture score that benefits from reduced multiple hypothesis testing for the admixture component of the score is an important open question that requires further consideration.

While this paper focuses on frequentist approaches for disease scoring in admixed populations, we mention that joint modeling of admixture and SNP association signals could be developed in a Bayesian framework [48]. For example, SNPs that lie in regions of high admixture signals could be given a higher prior of association with phenotype. We expect this type of approach to provide added value especially in regions with multiple independent causal variants in which region-based scores could yield increased signal over marginal SNP scores.

Although in this work we have focused on African Americans, in theory our approaches can be extended to other admixed populations such as Latino populations, which inherit ancestry from up to three continental ancestral (European, Native American and African) populations. The approaches presented in this work can be extended to three-way admixed populations either by considering one ancestry versus the rest strategy or by jointly modeling the three ancestry odds ratios so that a single SNP odds ratio would lead to implied ancestry odds ratios for each ancestry. However, we caution that in the context of Latino populations, more work is needed to assess the performance and possible biases of the local ancestry estimates and its potential effects on methods that incorporate admixture and case-control signals into disease scoring statistics.

A final consideration is in fine-mapping causal loci. Here the availability of samples—or chromosomal segments—of distinct ancestry is valuable [38] for localization of the causal variant. We note that the HET score could be used in localizing the causal variant under the hypothesis of no heterogeneity across populations; recent studies have provided empirical support for this hypothesis [49]. Importantly, by comparing MIX and SUM score the question whether the admixture signal is fully explained by the SNP odds ratio can be assessed. An important open question and future research direction is designing optimal algorithms for cross-population fine mapping that leverage the different LD patterns among the chromosomal segments of distinct ancestry.

## Methods

### Ethics statement

The CARe project has been approved by the Committee on the Use of Humans as Experimental Subjects (COUHES) of the Massachusetts Institute of Technology, and by the Institutional Review Boards of each of the nine parent cohorts.

### CARe data set

Affymetrix 6.0 genotyping and QC filtering of African-American samples from the CARe cardiovascular consortium was performed as described previously [25]. After QC filtering for each of ARIC, CARDIA, CFS, JHS and MESA cohorts and subsequent merging, 8,367 samples and 770,390 SNPs remained. To limit relatedness among samples we restricted all analyses to a subset of 6,209 samples in which all pairs have genome-wide relatedness of 0.10 or less (inferred using the *smartrel* program in EIGENSOFT 3.0; see Web Resources). We merged CARe genotype data with genotype data from the HapMap3 project [18]. HapMap3 samples had been genotyped on both Affymetrix 6.0 and Illumina 1M chips. We excluded SNPs that did not pass QC in HapMap3, as well as A/T and C/G SNPs to avoid allele complementarity issues, leaving 556,698 SNPs for further analysis. (We note that HAPMIX accuracy is insensitive to the number of SNPs, if at least 250,000 SNPs are used [23].)

### Inference of local ancestry using HAPMIX

When run in default mode, HAPMIX outputs local ancestry estimates as the expected probability of 0, 1 or 2 copies of European ancestry at each SNP (see ref. [23] and Web Resources). However, HAPMIX can also be run in a mode that outputs the inferred joint distribution of local ancestry and allele value, so as to resolve the “het-het” case (both genotype and local ancestry heterozygous). In order to obtain integer estimates of local ancestry, one approach is to simply round the probabilities, which however can lead to biased estimates in regions with limited SNP coverage. We chose an alternative approach that does not produce these types of biases: sampling from the probabilities for 0, 1 or 2 European chromosomes at each position. Results in this mode are highly concordant with the default mode, producing correlations of 100% in genome-wide ancestry and 98.8% in local ancestry.

### Simulated case-control phenotypes

We selected a random subset of 100,000 autosomal SNPs. For each SNP, we simulated phenotypes for *R* = 1.0 (null model) and *R* = 1.2,1.5,2.0 (causal models). For the null model, we chose random subsets of 1,000 cases and 1,000 controls. For causal models, we chose a random subset of 1,000 controls, and then chose 1,000 cases from the remaining samples so that samples with 0:1:2 reference alleles have relative probabilities 1:*R*:*R* ^2^ of being chosen.

### Association statistics for case-control phenotypes

#### ATT: the Armitage Trend Test

A χ^2^(1dof) statistic via the Armitage trend test with adjustment for genome-wide ancestry, as described previously [50]. Genome-wide ancestry was inferred as the genome-wide average of local ancestry estimates from HAPMIX [23]. We note that this is >99% correlated to the top eigenvector of a principal components analysis run using CEU and YRI from HapMap3 to compute principal components [18], [20].

#### SNP1: SNP association conditioned on local ancestry

A χ^2^(1dof) likelihood ratio test that compares the null hypothesis of case-control odds ratio R = 1 with the alternate hypothesis of R ≠1, where R is assumed to be the same across populations, while the allele frequencies are treated as nuisance parameters.

For every local ancestry *X*_1_ *X*_2_ (AA, AE, or EE) and phenotype *Y* (1 for cases, 0 for controls), let 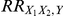, 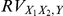,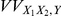 denote the counts of individuals with genotypes 2, 1 or 0. Then the SNP1 likelihood is defined as
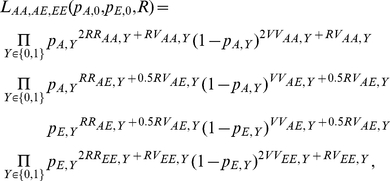
where 
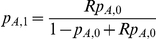
, 
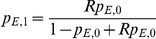
 represent the allele frequencies in cases, 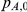, 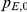 represent allele frequencies in controls and R is the allelic odds ratio.

Then, the χ^2^ statistic with 1 degree of freedom is:
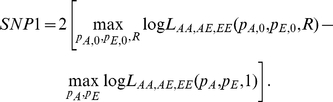



#### ADM: admixture association using cases only

A χ^2^(1dof) likelihood ratio test that compares the local ancestry in the disease cases to the average local ancestry across the genome in the same disease cases. This is more powerful than comparing cases to controls, since no statistical noise is introduced from controls [7]. However, it is critical when using this approach to infer local ancestry using a method that has been shown not to produce artifactual deviations in average local ancestry in large data sets of controls from the admixed population [23].

Let *θ_i_* be the genome-wide ancestry of individual *i*, and let *N_i_* be the number of European chromosomes in individual *i* at the candidate SNP. Restricting to disease cases only, we define the likelihood as function of ancestry odds ratio *Ω*, where *Ω* is the multiplicative risk for disease given one or two European alleles. It follows that the likelihood for 2, 1, or 0 European alleles at individual i is:



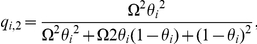


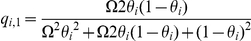


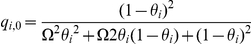



Then the likelihood is 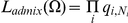, with the a χ^2^(1dof) likelihood ratio test defined as: 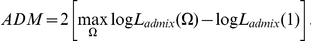



#### SUM: sum of SNP1 and ADM

A χ^2^(2dof) that sums the SNP1 and the ADM statistics [25]. We note that, since SNP1 conditions out the local ancestry and the ADM statistic employs only the local ancestry, these two tests contain independent information.

#### MIX: mixed SNP and admixture association

A χ2(1dof) test that combines the SNP1 and ADM likelihood functions by using the implied ancestry odds ratio 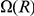 under the assumption of a single causal variant with same odds ratio R across the European and African populations.

The MIX likelihood is defined as the product of the likelihoods for SNP1 and ADM as 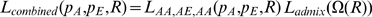, where 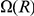
is the relative increase in risk per extra European allele under the assumption of single causal variant with odds ratio R. It follows that 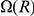 is a function of the SNP odds ratio *R* and the population allele frequencies in controls: 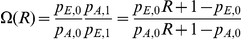. We then compute a χ^2^ statistic with 1 degree of freedom as:
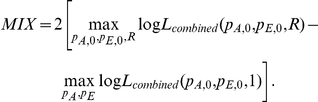



#### HET: test for heterogeneity of effect size as a function of local ancestry

A χ2(1dof) test that compares the alternate hypothesis of different odds ratios in different ancestries with the null model that assumes the same odds ratio. The likelihood 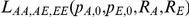 extends the SNP1 likelihood by allowing ancestry specific odds ratios (R_A_ and R_E_) which leads to 
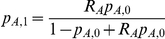
 and 
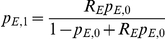
. We then compute a χ^2^(1dof) statistic as: 
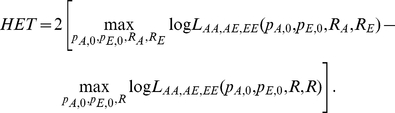



### Incorporating the empirical variance of the average ancestry estimates in ADM and MIX scores

We incorporate the observed variance of the average local ancestry across the genome assuming that the average local ancestry 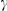
at each SNP is normally distributed with mean 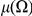 and standard deviation 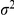, where 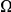 is the ancestry odds ratio. We estimate 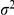 empirically and set 
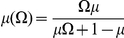
, where 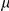 is the empirical mean across the genome of the per SNP average local ancestry estimates. Then, the admixture likelihood becomes 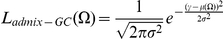. We can then compute a χ^2^(1dof) statistic, ADMGC, that incorporates the empirical variance and in the ADM score as:
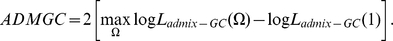
In a similar manner we can replace 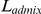
with 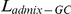
in the admixture component of the MIX likelihood to compute a new χ^2^(1dof) statistic MIXGC, that incorporates the empirical variance of the average local ancestry:
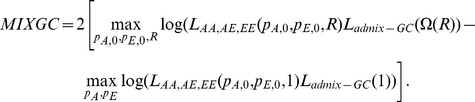



### Optimization algorithm for association statistics for case-control phenotypes

Many of the likelihoods defined above require a multidimensional optimization. The number of parameters optimized in the likelihoods is 3 for the SNP1 score, 1 for the ADM score, 3 for the MIX score and 4 for the HET score. (The HET score can be reduced to two independent 2-parameter optimizations by considering cases and controls separately.) For the ADM score, Newton’s method was used. For the SNP1, MIX and HET scores, Brent’s algorithm was used (GSL software library implementation; see Web Resources). The maximization is performed in one dimension over each parameter in turn, repeating for each parameter until the algorithm converges. In rare instances, extreme variation in the slope of the log likelihood as a function of odds ratio can cause the algorithm to not converge; in this situations a simple binary search is used.

### Genotype imputation in African Americans

We employed the widely used MACH [51] imputation method to infer genotypes at untyped SNPs in the CARe African American samples. As reference haplotypes we used either the cosmopolitan approach of providing all the CEU and YRI haplotypes from HapMap Phase 3 data [18], or a local ancestry aware approach in which, for every locus in every sample, we provided either YRI, CEU+YRI, CEU reference haplotypes to MACH according to the number of copies of YRI (2/1/0) inferred by HAPMIX. We note that the local ancestry aware approach has been previously shown to boost imputation accuracy in admixed populations [24. 35]. For both strategies we ran MACH in two steps, first by training the model parameters on a random sample of 200 individuals with the rounds parameter set to 50 followed by imputation of all the samples using the trained model from step 1. Importantly, we note that the local ancestry aware approach can be applied as an add-on to any imputation method.

### Accounting for different imputation quality in African and European segments

Even when the true odds ratio is the same across populations, different imputation quality across the segments with different ancestries can lead to different estimates for the allelic odds ratios in European versus African segments. We account for this by adjusting the observed allelic odds ratios in the SNP1 and the MIX scores as follows. Following a derivation similar to [52] (see Text S1) we show that the expected observed odds ratio at an imputed causal SNP with true odds ratio R, is a function of R, the imputation accuracy (as measured by the correlation between true and imputed SNP), and the allele frequency: 
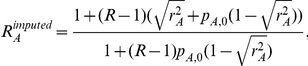


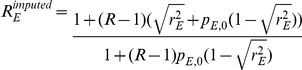
Unfortunately we do not know the true genotypes, and thus cannot compute the correlation between the true and imputed genotypes. However, reliable estimates for this correlation have been proposed; here we chose to use MACH 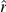 estimates shown to produce robust estimates of imputation quality [53]. To estimate ancestry-specific imputation error rates, we restrict the computation to segments containing both alleles from that ancestry. Given that imputation accuracies are estimated directly from the data, 
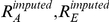
 depend on the term R and the allele frequencies. Then, the likelihood term from the MIX admixture association score becomes 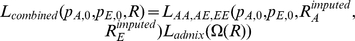. As in the previous version of the score, the optimization is done over the three free terms 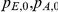 and 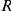. SNP1 score is updated in a similar fashion.

### Application of the scores when the causal SNP is not typed or imputed

We randomly selected 100,000 autosomal SNPs and simulated phenotypes as described above using R = 1.5. For all the compared scores, we computed the maximum statistic over all SNP across a region centered on the SNP of interest (taking the 20 SNPs upstream and 20 SNPs downstream). We computed the maximum of the statistics either over 41 SNPs by including the simulated causal SNP or over 40 SNPs by ignoring the statistics at the simulated causal SNP.

### Application to coronary heart disease and type 2 diabetes case-control phenotypes

Case-control phenotypes for coronary heart disease (CHD) and type 2 diabetes (T2D) were ascertained as described previously [25]. In each case, phenotypes were available for only a subset of the five CARe cohorts. Restricting to 6,209 unrelated individuals as defined above, we analyzed 929 cases and 4,150 controls for T2D, and 179 cases and 3,328 controls for CHD. For every analyzed SNP we performed imputation within a region of 10Mb centered on the SNP of interest using the MACH imputation method under the local ancestry aware framework. We assessed the scoring statistics at all SNPs within 100Kb of the SNPs of interest.

### Application to FGFR2 locus

The FGFR2 locus has been associated with breast cancer in women of European and Asian descent [36], and further fine mapping in African-American women has identified SNP rs2981578 as showing the highest signal of association [36], [37]. We analyzed data from a GWAS including 5,761 unrelated African-American women from 11 epidemiological studies: The Multiethnic Cohort Study (MEC) [54], The Los Angeles component of The Women’s Contraceptive and Reproductive Experiences (CARE) cohort [55], The Women’s Circle of Health Study (WCHS) [56], The San Francisco Bay Area Breast Cancer Study (SFBC) [57], The Northern California Breast Cancer Family Registry (NC-BCFR) [58], [59], The Carolina Breast Cancer Study (CBCS) [60], The Prostate, Lung, Colorectal, and Ovarian Cancer Screening Trial (PLCO) [61], The Nashville Breast Health Study (NBHS)[62], The Wake Forest University Breast Cancer Study (WFBC) [63]. Informed consent was obtained from all subjects. Detailed information about the design and organization of each study will be provided elsewhere (C. Haiman and colleagues, unpublished data). Genotyping was conducted using the Illumina Human1M-Duo BeadChip. A total of 1,043,036 SNPs were kept after QC filtering. Imputation was performed using the MACH software, providing as reference all the haplotypes of CEU and YRI HapMap Phase 2 panels). We focused our analysis on all the typed or imputed SNPs, 251 in total, located 100Kb upstream and downstream of SNP rs2981578.

### Simulated quantitative phenotypes

For each of 100,000 autosomal SNPs, we simulated phenotypes for *ε* = 0 (null model) and *ε* = 0.05,0.10,0.20 (causal model), using a random subset of 2,000 samples. For the null model, phenotypes were sampled from a normal distribution with mean 0 and variance 1. For the causal model, the mean was shifted to 0:*ε*:2*ε* for 0:1:2 reference alleles. In each case, we subtracted out the overall phenotypic mean.

### Association statistics for quantitative phenotypes

#### Armitage Trend Test (QATT), χ^2^(1dof)

Let 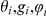 denote genome-wide ancestry, genotype (0, 1 or 2) and phenotype for sample *i*. The model is 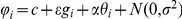. We compute a χ^2^(1dof) statistic as 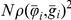, where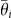 is adjusted to mean 0 and 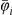 and 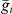 are each adjusted for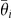. We also compute the effect size *ε*.

#### SNP association conditioned on local ancestry (QSNP1), χ^2^(1dof)

Let 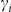 denote local ancestry (0, 1 or 2 European copies) for sample *i*. The model is 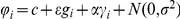. We compute a χ^2^(1dof) statistic as 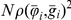, where 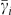 is adjusted to mean 0 and 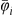 and 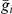 are each adjusted for 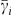. We also compute the effect size *ε*.

#### Admixture association (QADM), χ^2^(1dof)

The model is 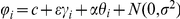. We compute a χ^2^(1dof) statistic as 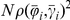, where 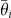 is adjusted to mean 0 and 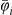 and 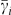 are each adjusted for 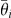. We also compute the effect size *ε*.

#### Sum of QSNP1 and QADM (QSUM), χ^2^(2dof)

We sum the two χ^2^(1dof) statistics to produce a χ^2^(2dof) statistic.

#### Test for heterogeneity of effect size as a function of local ancestry (QHET), χ^2^(1dof)

Let 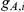 and 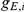
denote the number of reference alleles of African or European local ancestry. If joint local ancestry and phasing information is not available and 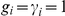, we use expected values 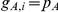 and 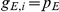, where 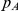 and 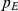 are estimated as above by maximizing 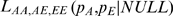. The model is 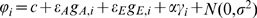. We compute a χ^2^(2dof) statistic as *N* times the proportion of variance of 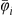 jointly predicted by 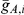 and 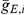, where 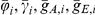 are adjusted to mean 0 and 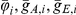 are adjusted for 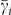. We also compute the effect sizes 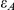 and 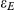. We define the *QHET* score as the χ^2^(2dof) statistic minus the *QSNP1* score, thus testing the statistical significance of 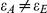.

### Application to LDL and HDL cholesterol phenotypes

LDL and HDL cholesterol phenotypes in CARe samples were ascertained as described previously. We analyzed 5,801 samples for LDL and 5,946 samples for HDL for which phenotypic data were available, restricting to 6,209 unrelated individuals as defined above. For every analyzed SNP we performed imputation within a region of 10Mb centered on the SNP of interest using the MACH imputation method under the local ancestry aware framework. We assessed the scoring statistics at all SNPs within 100Kb of the SNPs of interest.

### Web resources

http://www.hsph.harvard.edu/faculty/alkes-price/software/(MIXSCORE software)

http://www.hsph.harvard.edu/faculty/alkes-price/software/(EIGENSOFT software)

http://www.stats.ox.ac.uk/~myers/software.html (HAPMIX software)

## Supporting Information

Figure S1 Principal components analysis of CARe and HapMap3 samples. Only the HapMap3 populations CEU, YRI and CHB were used to compute principal components.
(0.11 MB PDF)

Figure S2 Average local ancestry of 6,209 CARe samples.
(0.07 MB PDF)

Figure S3 Proportion of SNPs with imputation accuracy difference in European versus African segments under a specified threshold. The imputation accuracy in European (African) segments was estimated for each SNP as the squared correlation between true masked genotypes and imputed genotypes restricted to samples containing 2(0) European (African) alleles at that locus.
(0.04 MB PDF)

Figure S4 Proportion of SNPs achieving genome-wide significance as function of the expected difference in odds ratios between Africans and Europeans. Scores were computed at SNPs neighboring 100,000 simulated causal SNPs (R = 1.5), tagging with different LD in European versus Africans the simulated causal.
(0.03 MB PDF)

Table S1 Average value and statistical power of simulated case-control MIX score in African Americans imputed genotypes under various imputation settings (MIX*-denotes no adjustment for differences in imputation error rates). For each setting we list the average χ^2^ value and proportion of SNPs for which the score attains genome-wide significance (defined as P<5e-08), for random SNPs as well as SNPs in the top decile of population differences (Δ>0.4), for *R* = 1.0, *R* = 1.2, *R* = 1.5, *R* = 2.0 simulations (see main text). The proportion of SNPs attaining genome-wide significance is indicated in parentheses. Adjusting for imputation quality difference improves the power in all cases. Local ancestry aware imputation yields increase in power. Overall, the MIX score with local ancestry aware imputation and adjustment for differences in imputation quality yields the best results.
(0.03 MB DOC)

Table S2 Average statistic and statistical power of case-control scores in African Americans computed for different number of cases and R = 1.5. The number of controls is set to 1000. For each score we list the average χ^2^ value and proportion of SNPs for which the score attains genome-wide significance (defined as P<5e-08 for all scores except ADM, P<1e-05 for ADM). In general all the scores show decrease in performance with the decrease in number of cases. The increase in performance of MIX over ATT score diminishes with the number of cases: for 100 cases, the increase of average χ^2^ in MIX over ATT is less than 1%, while for 1000 cases, the same increase is greater than 5%.
(0.03 MB DOC)

Table S3 Average statistic and statistical power of case-control scores in African Americans computed under various disease models. 1000 cases and 1000 controls were simulated at 100,000 SNPs with odds ratio R. For each score we list the average χ^2^ value and proportion of SNPs for which the score attains genome-wide significance (defined as P<5e-08 for all scores except ADM, P<1e-05 for ADM). In the multiple causal scenarios, for each of the 100,000 SNPs, a nearby SNP (distance less than 5Mb and with r^2^<0.1) was selected and a disease model with two causal SNPs was simulated in which both SNPs had an odds ratio of 1.5. With the exception of the ‘Dominant’ scenario in which ATT and MIX obtain similar results, in all remaining cases MIX outperforms the other scores in terms of power.
(0.04 MB DOC)

Table S4 Results for LDL and HDL quantitative phenotypes. (a) We list results for each score (-log in base 10 of the p-value) for genotyped SNPs that have previously been associated to LDL in CARe samples, the imputed (* denotes imputed SNPs) or genotyped SNPs producing the most significant P-values, and the best score for each of the five scores. (b) Analogous to (a), for SNPs associated to HDL. The value achieving the smallest p-value is denoted in bold.
(0.08 MB DOC)

Text S1 Supplementary Note.
(0.09 MB DOC)
