# Supplementary figures and images for: Enhanced Statistical Tests for GWAS in Admixed Populations: Assessment using African Americans from CARe and a Breast Cancer Consortium
Source: PLoS Genet. 2011 Apr 21;7(4):e1001371. doi: 10.1371/journal.pgen.1001371 (PMC3080860; doi:10.1371/journal.pgen.1001371)

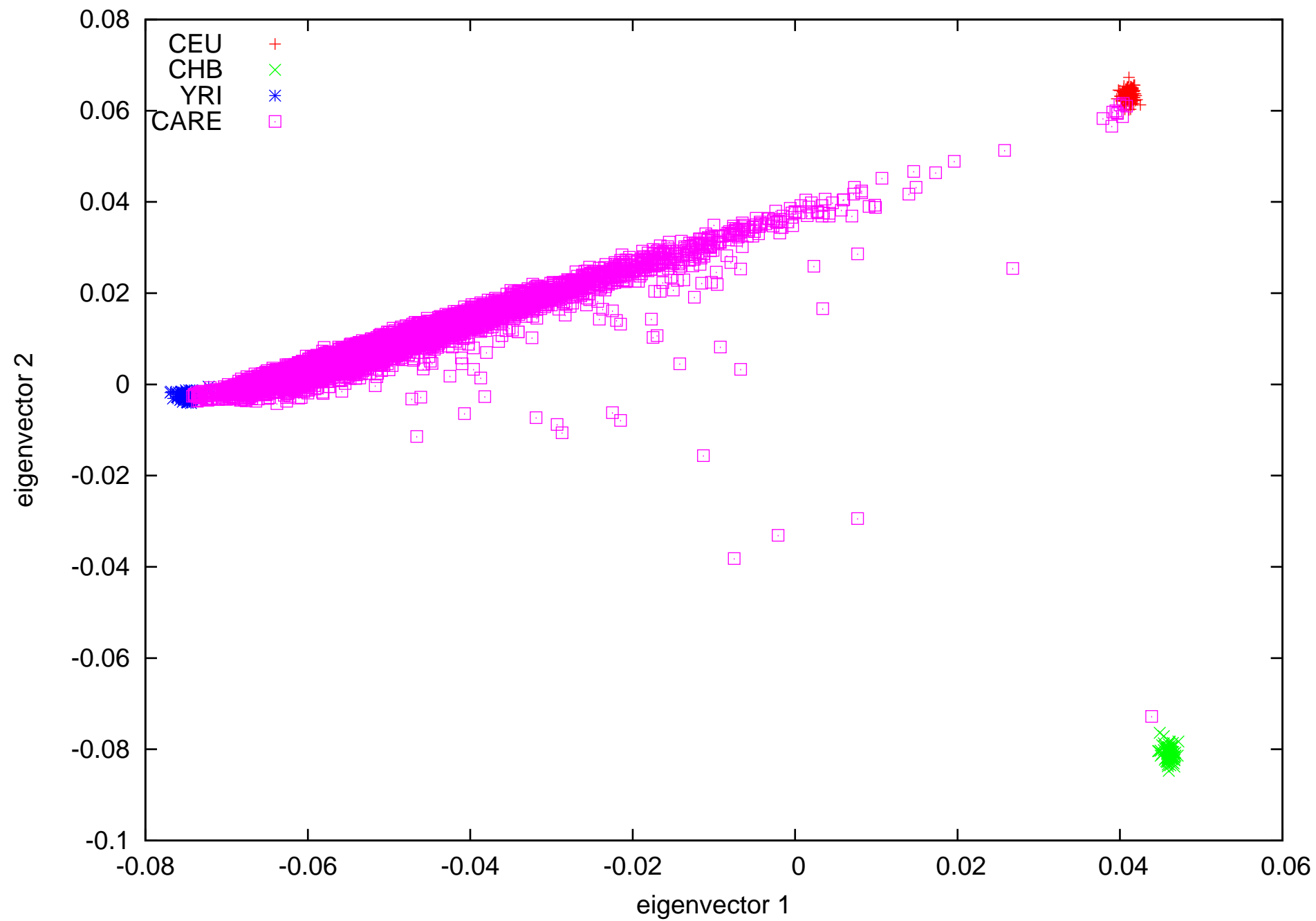

Supplement: Figure S1 — Principal components analysis of CARe and HapMap3 samples. Only the HapMap3 populations CEU, YRI and CHB were used to compute principal components. (0.11 MB PDF) [file pgen.1001371.s001.pdf]

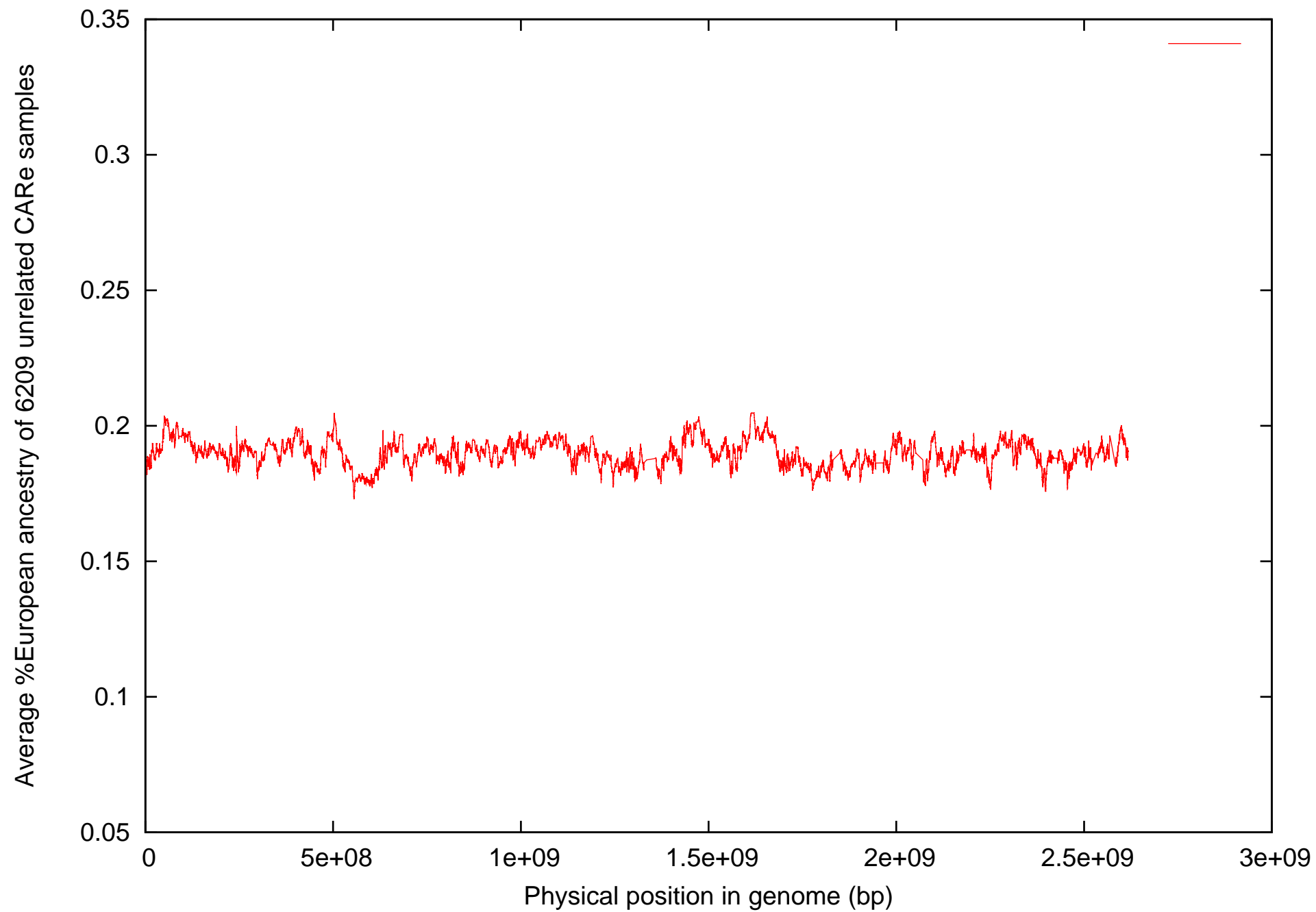

Supplement: Figure S2 — Average local ancestry of 6,209 CARe samples. (0.07 MB PDF) [file pgen.1001371.s002.pdf]

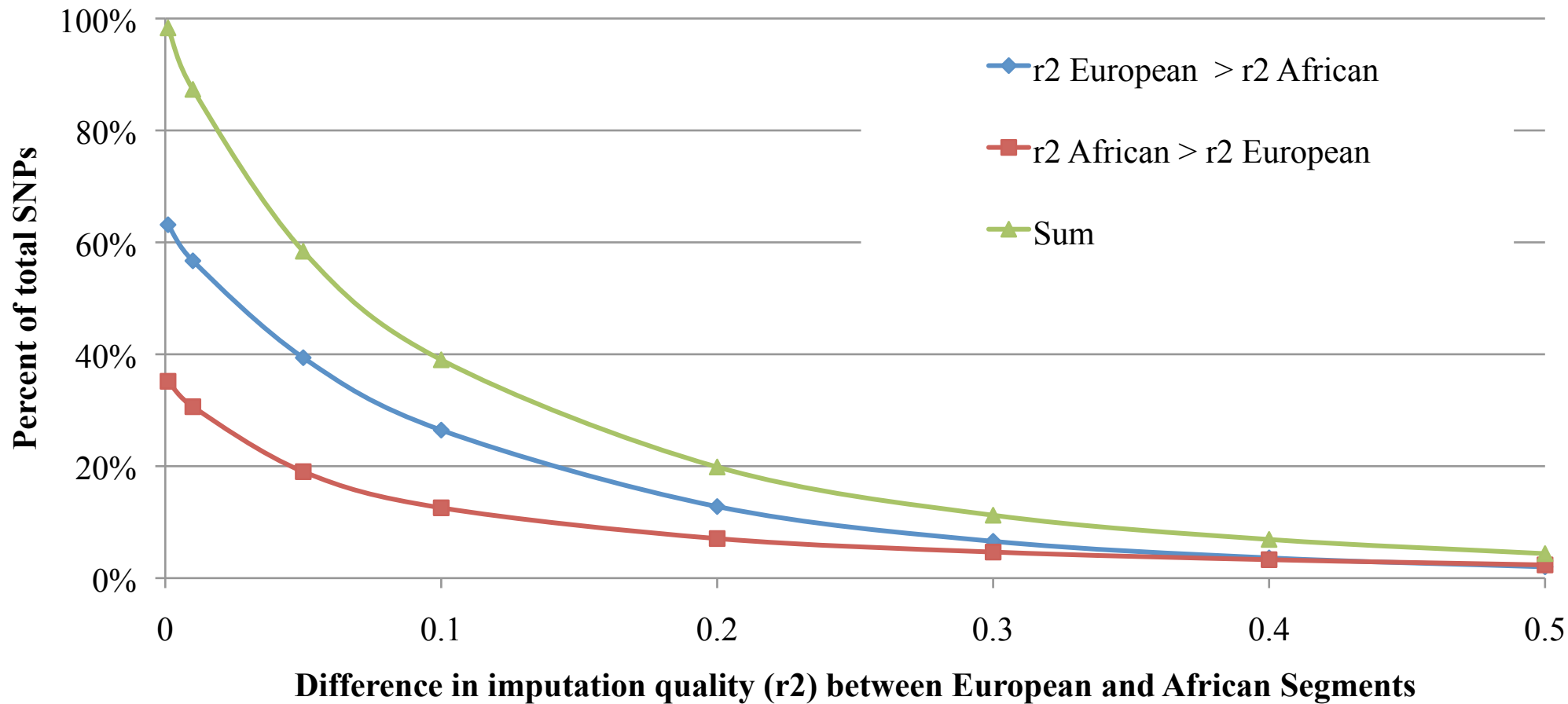

Supplement: Figure S3 — Proportion of SNPs with imputation accuracy difference in European versus African segments under a specified threshold. The imputation accuracy in European (African) segments was estimated for each SNP as the squared correlation between true masked genotypes and imputed genotypes restricted to samples containing 2(0) European (African) alleles at that locus. (0.04 MB PDF) [file pgen.1001371.s003.pdf]

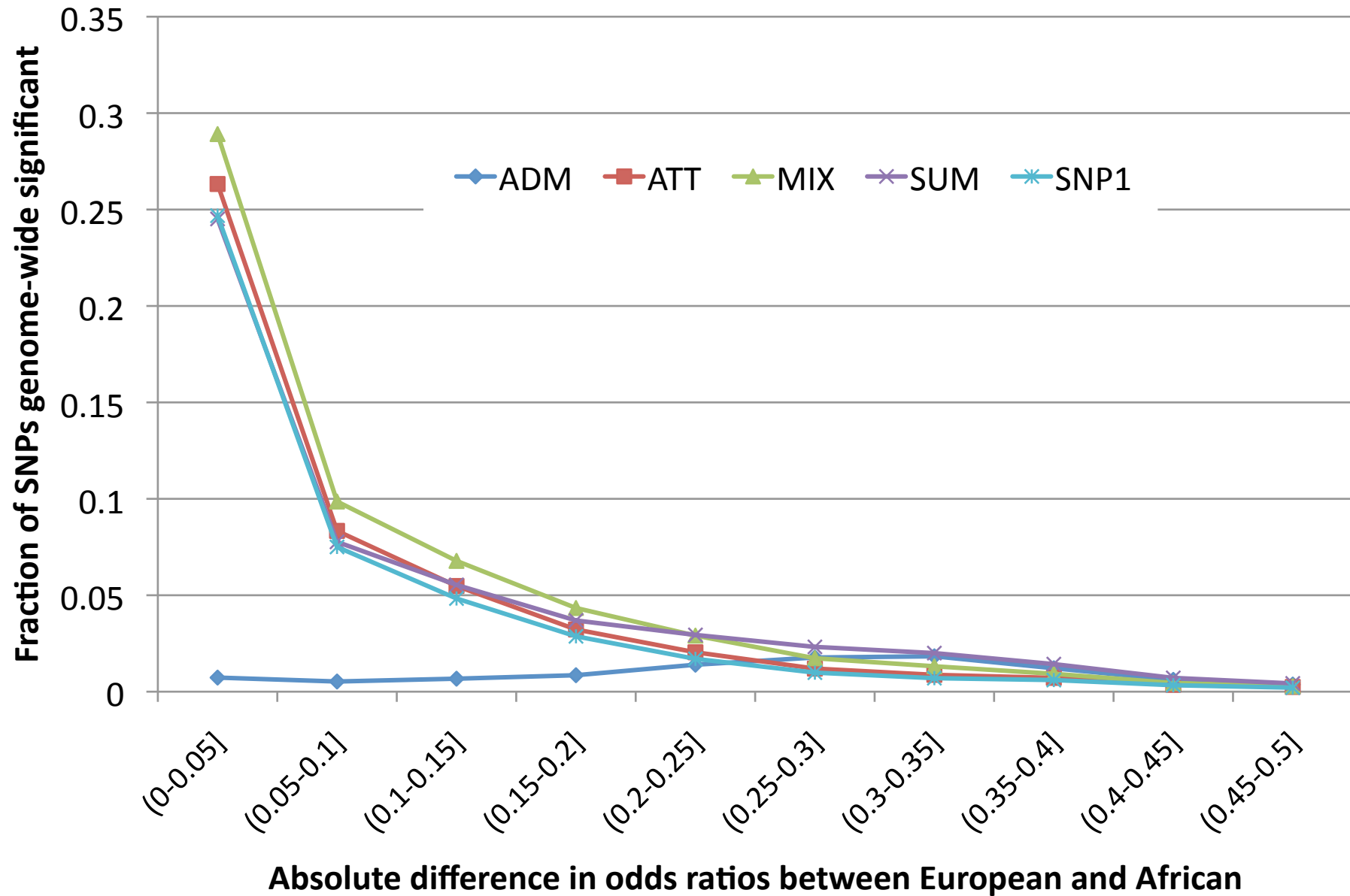

Supplement: Figure S4 — Proportion of SNPs achieving genome-wide significance as function of the expected difference in odds ratios between Africans and Europeans. Scores were computed at SNPs neighboring 100,000 simulated causal SNPs (R = 1.5), tagging with different LD in European versus Africans the simulated causal. (0.03 MB PDF) [file pgen.1001371.s004.pdf]
