# Supplementary material for: Enhanced Statistical Tests for GWAS in Admixed Populations: Assessment using African Americans from CARe and a Breast Cancer Consortium
Source: PLoS Genet. 2011 Apr 21;7(4):e1001371. doi: 10.1371/journal.pgen.1001371 (PMC3080860; doi:10.1371/journal.pgen.1001371)
Supplement: Table S1 — Average value and statistical power of simulated case-control MIX score in African Americans imputed genotypes under various imputation settings (MIX*-denotes no adjustment for differences in imputation error rates). For each setting we list the average χ2 value and proportion of SNPs for which the score attains genome-wide significance (defined as P<5e-08), for random SNPs as well as SNPs in the top decile of population differences (Δ>0.4), for R = 1.0, R = 1.2, R = 1.5, R = 2.0 simulations (see main text). The proportion of SNPs attaining genome-wide significance is indicated in parentheses. Adjusting for imputation quality difference improves the power in all cases. Local ancestry aware imputation yields increase in power. Overall, the MIX score with local ancestry aware imputation and adjustment for differences in imputation quality yields the best results. (0.03 MB DOC) [file pgen.1001371.s005.doc]

|  | Imputation  Reference  Panel | R=1.0 random | R=1.0 Δ>0.4 | R=1.2 random | R=1.2 Δ>0.4 | R=1.5 random | R=1.5 Δ>0.4 | R=2.0 random | R=2.0 Δ>0.4 |
| --- | --- | --- | --- | --- | --- | --- | --- | --- | --- |
| MIX* χ2(1dof) | CEU+YRI | 1.00 (0.000) | 1.05 (0.000) | 5.48 (0.001) | 7.64 (0.003) | 23.11 (0.316) | 33.52 (0.579) | 64.96 (0.776) | 94.35 (0.974) |
| MIX  χ2(1dof) | CEU+YRI | 1.00 (0.000) | 1.06 (0.000) | 5.48 (0.001) | 7.66 (0.003) | 23.13 (0.316) | 33.63 (0.581) | 65.00 (0.776) | 94.65 (0.975) |
| MIX*  χ2(1dof) | Local ancestry [CEU+YRI] | 1.00 (0.000) | 1.05 (0.000) | 5.49 (0.001) | 7.72 (0.003) | 23.19 (0.318) | 33.92 (0.588) | 65.14 (0.778) | 95.31 (0.977) |
| MIX χ2(1dof) | Local ancestry [CEU+YRI] | 1.01 (0.000) | 1.06 (0.000) | 5.50 (0.001) | 7.74 (0.003) | 23.21 (0.318) | 34.04 (0.591) | 65.19 (0.778) | 95.61 (0.979) |
