# Supplementary material for: Enhanced Statistical Tests for GWAS in Admixed Populations: Assessment using African Americans from CARe and a Breast Cancer Consortium
Source: PLoS Genet. 2011 Apr 21;7(4):e1001371. doi: 10.1371/journal.pgen.1001371 (PMC3080860; doi:10.1371/journal.pgen.1001371)
Supplement: Table S2 — Average statistic and statistical power of case-control scores in African Americans computed for different number of cases and R = 1.5. The number of controls is set to 1000. For each score we list the average χ2 value and proportion of SNPs for which the score attains genome-wide significance (defined as P<5e-08 for all scores except ADM, P<1e-05 for ADM). In general all the scores show decrease in performance with the decrease in number of cases. The increase in performance of MIX over ATT score diminishes with the number of cases: for 100 cases, the increase of average χ2 in MIX over ATT is less than 1%, while for 1000 cases, the same increase is greater than 5%. (0.03 MB DOC) [file pgen.1001371.s006.doc]

|  | 100 Cases | 200 Cases | 500 Cases | 1000 Cases |
| --- | --- | --- | --- | --- |
| ATT | 5.54  (0.001) | 9.29  (0.013) | 17.47  (0.146) | 25.73  (0.380) |
| SNP1 | 5.37  (0.001) | 9.01  (0.011) | 16.99  (0.135) | 25.10  (0.363) |
| ADM | 1.171  (0.000) | 1.390  (0.000) | 2.032  (0.0001) | 3.14  (0.008) |
| SUM | 6.54  (0.001) | 10.40  (0.007) | 19.02  (0.116) | 28.23  (0.356) |
| MIX | 5.57  (0.001) | 9.42  (0.014) | 18.01  (0.160) | 27.08  (0.413) |
