# Supplementary material for: Enhanced Statistical Tests for GWAS in Admixed Populations: Assessment using African Americans from CARe and a Breast Cancer Consortium
Source: PLoS Genet. 2011 Apr 21;7(4):e1001371. doi: 10.1371/journal.pgen.1001371 (PMC3080860; doi:10.1371/journal.pgen.1001371)
Supplement: Table S3 — Average statistic and statistical power of case-control scores in African Americans computed under various disease models. 1000 cases and 1000 controls were simulated at 100,000 SNPs with odds ratio R. For each score we list the average χ2 value and proportion of SNPs for which the score attains genome-wide significance (defined as P<5e-08 for all scores except ADM, P<1e-05 for ADM). In the multiple causal scenarios, for each of the 100,000 SNPs, a nearby SNP (distance less than 5Mb and with r2<0.1) was selected and a disease model with two causal SNPs was simulated in which both SNPs had an odds ratio of 1.5. With the exception of the ‘Dominant’ scenario in which ATT and MIX obtain similar results, in all remaining cases MIX outperforms the other scores in terms of power. (0.04 MB DOC) [file pgen.1001371.s007.doc]

|  | 1 causal SNP  (R=1.5) | Dominant (R=2.0) | Recessive  (R=2.0) | 2 causal SNPs  (R=1.5) | 2 causal SNPs  (R=1.5, Δ>0.4) |
| --- | --- | --- | --- | --- | --- |
| ATT | 25.73 (0.380) | 22.92  (0.370) | 24.07  (0.382) | 26.67  (0.401) | 31.39  (0.520) |
| SNP1 | 25.10 (0.363) | 20.84  (0.330) | 24.89  (0.393) | 26.00  (0.384) | 28.65  (0.450) |
| ADM | 3.14 (0.008) | 2.99  (0.013) | 2.93  (0.009) | 4.91  (0.033) | 12.53  (0.164) |
| SUM | 28.23 (0.356) | 23.83  (0.332) | 27.81  (0.387) | 30.91  (0.406) | 41.18  (0.615) |
| MIX | 27.08 (0.413) | 22.64  (0.366) | 26.68  (0.423) | 28.18  (0.434) | 35.30  (0.590) |
