# Supplementary material for: Enhanced Statistical Tests for GWAS in Admixed Populations: Assessment using African Americans from CARe and a Breast Cancer Consortium
Source: PLoS Genet. 2011 Apr 21;7(4):e1001371. doi: 10.1371/journal.pgen.1001371 (PMC3080860; doi:10.1371/journal.pgen.1001371)
Supplement: Table S4 — Results for LDL and HDL quantitative phenotypes. (a) We list results for each score (-log in base 10 of the p-value) for genotyped SNPs that have previously been associated to LDL in CARe samples, the imputed (* denotes imputed SNPs) or genotyped SNPs producing the most significant P-values, and the best score for each of the five scores. (b) Analogous to (a), for SNPs associated to HDL. The value achieving the smallest p-value is denoted in bold. (0.08 MB DOC) [file pgen.1001371.s008.doc]

Table S4(a)

| SNP | chrom | position (build36) | CEU  freq | YRI  freq | QATT | QSNP1 | QADM | QSUM | QHET |
| --- | --- | --- | --- | --- | --- | --- | --- | --- | --- |
| rs629301 | 1 | 109,530,348 | 0.30 | 0.27 | 10.93 | 10.45 | 1.19 | 10.27 | 1.84 |
| rs12740374* | 1 | 109,529,632 | 0.30 | 0.18 | 13.03 | 13.18 | 1.2 | 12.95 | 2.06 |
| Best Score | - | - | - | - | 13.03 | **13.18** | 1.84 | 12.95 | - |
| rs562338 | 2 | 21,199,973 | 0.78 | 0.24 | 4.88 | 2.82 | 3.46 | 4.96 | 0.73 |
| rs568938* | 2 | 21,215,268 | 0.75 | 0.25 | 6.72 | 4.31 | 3.92 | 6.79 | 0.68 |
| Best Score | - | - | - | - | 6.72 | 4.43 | 4.19 | **6.79** | - |
| rs6728440 | 2 | 19,920,974 | 0.13 | 0.00 | 1.06 | 2.12 | 1.67 | 2.7 | 0.07 |
| rs1866183* | 2 | 19,944,727 | 0.00 | 0.23 | 4.99 | 4.36 | 1.69 | 4.79 | 0.07 |
| Best Score | - | - | - | - | **4.99** | 4.37 | 2.29 | 4.79 | - |
| rs7560236 | 2 | 22,988,435 | 0.00 | 0.08 | 3.44 | 4.21 | 4.18 | 6.94 | 0.33 |
| rs2001795* | 2 | 23,027,188 | 0.42 | 0.44 | 2.86 | 2.76 | 3.5 | 4.95 | 0.04 |
| Best Score | - | - | - | - | 3.44 | 4.21 | 4.42 | **6.94** | - |
| rs9306885 | 2 | 19,910,460 | 0.72 | 0.16 | 0.83 | 2.94 | 1.64 | 3.42 | 0.43 |
| rs1866182* | 2 | 19,944,611 | 0.00 | 0.23 | 4.92 | 4.37 | 1.42 | 4.57 | 0.25 |
| Best Score | - | - | - | - | **4.92** | 4.37 | 2.21 | 4.57 | - |
| rs17441606 | 2 | 19,490,063 | 0.33 | 0.11 | 2.33 | 2.95 | 1.47 | 3.28 | 0.18 |
| rs7603289* | 2 | 19,504,403 | 0.67 | 0.75 | 2.95 | 3.31 | 1.66 | 3.78 | 0.12 |
| Best Score | - | - | - | - | 2.95 | 3.31 | 2.17 | **3.78** | - |
| rs5929 | 19 | 11,087,800 | 0.01 | 0.09 | 4.55 | 4.35 | 0.54 | 3.86 | 0.11 |
| rs8107532* | 19 | 11,116,993 | 0.00 | 0.09 | 4.79 | 4.76 | 0.38 | 4.15 | 0.27 |
| Best Score | - | - | - | - | **4.79** | 4.77 | 0.79 | 4.23 | - |

Table S4(b)

| SNP | chrom | position (build36) | CEU  freq | YRI  freq | QATT | QSNP1 | QADM | QSUM | QHET |
| --- | --- | --- | --- | --- | --- | --- | --- | --- | --- |
| rs4846914 | 1 | 226,602,426 | 0.58 | 0.01 | 3.05 | 1.8 | 1.78 | 2.51 | 0.49 |
| rs2281719 | 1 | 226,604,394 | 0.59 | 0.01 | 3.07 | 1.8 | 1.77 | 2.5 | 0.28 |
| Best Score | - | - | - | - | **3.07** | 1.81 | 2.46 | 2.74 | - |
| rs6748157 | 2 | 28,645,012 | 0.49 | 0.03 | 2.12 | 0.71 | 2.52 | 2.27 | 0.51 |
| rs6547861* | 2 | 28,742,922 | 0.00 | 0.19 | 1.66 | 2.56 | 2.95 | 4.25 | 2.14 |
| Best Score | - | - | - | - | 3.02 | 2.56 | 3.68 | **4.25** | - |
| rs17482753 | 8 | 19,876,926 | 0.13 | 0.03 | 2.57 | 2.68 | 0.92 | 2.58 | 1.04 |
| rs10096633* | 8 | 19,875,201 | 0.13 | 0.48 | 7.5 | 6.59 | 1.36 | 6.65 | 0.17 |
| Best Score | - | - | - | - | **7.5** | 6.75 | 1.48 | 6.65 | - |
| rs255052 | 16 | 66,582,496 | 0.14 | 0.25 | 7.14 | 6.94 | 0.57 | 6.37 | 0.47 |
| rs16942887* | 16 | 66,485,543 | 0.90 | 0.70 | 7.62 | 7.32 | 0.72 | 6.85 | 0.41 |
| Best Score | - | - | - | - | **7.62** | 7.32 | 0.97 | 6.85 | - |
| rs2271293 | 16 | 66,459,571 | 0.10 | 0.07 | 4.37 | 4.55 | 0.72 | 4.18 | 0.02 |
| rs16942887* | 16 | 66,485,543 | 0.90 | 0.70 | 7.64 | 7.37 | 0.61 | 6.82 | 0.48 |
| Best Score | - | - | - | - | **7.64** | 7.37 | 1.05 | 6.82 | - |
| rs2967605 | 19 | 8,375,738 | 0.18 | 0.24 | 1.45 | 1.42 | 0.44 | 1.11 | 0.02 |
| rs2913968* | 19 | 8373235 | 0.708333 | 0.641667 | 2.53 | 2.57 | 0.56 | 2.22 | 0.17 |
| Best Score | - |  | - | - | 2.53 | **2.57** | 0.97 | 2.22 | - |
