# Supplementary material for: Enhanced Statistical Tests for GWAS in Admixed Populations: Assessment using African Americans from CARe and a Breast Cancer Consortium
Source: PLoS Genet. 2011 Apr 21;7(4):e1001371. doi: 10.1371/journal.pgen.1001371 (PMC3080860; doi:10.1371/journal.pgen.1001371)
Supplement: Text S1 — Supplementary Note. (0.09 MB DOC) [file pgen.1001371.s009.doc]

Text S1

SNP1 score is analogous to disease mapping in European or African populations

The SNP1 score is analogous to disease mapping in European or African populations, because it is conditioned on local ancestry. We verified this intuitive observation by performing two simulations. First, we repeated our R=1.5 simulation using only African-American samples that had 2 copies of African ancestry inferred by HAPMIX, thus effectively simulating samples of 100% African ancestry. We obtained an average Armitage Trend Test χ^2^(1dof) score of 25.2, which is very similar to the value of 25.0 for the SNP1 score. Second, we verified that simulations of 100% African and 100% European individuals obtained χ^2^(1dof) scores that were nearly identical to each other (23.1 and 22.8; both values were lower than 25.2 because simulations were based on HapMap frequencies, which contain additional sampling noise).

Fully powered association statistics do not require joint local ancestry inference/phasing

We investigated whether joint local ancestry inference and phasing information—which in the case of heterozygous genotype and heterozygous local ancestry will resolve which allele is on a European chromosome and which allele is on an African chromosome—could improve upon the MIX score. We ran HAPMIX in a mode which outputs joint local ancestry and phasing information, and implemented a variant of the MIX score that uses this information. Letting *R* and *V* denote ref and var allele counts, *A* and *E* denote African or European local ancestry, and Y denote phenotype (1 for case, 0 for control), we have:

,

,

We determined that this score produced values very similar to the MIX score, with an average value between 0.999 and 1.001 times the value of the MIX score for each column in Table 1. Thus, inclusion of joint local ancestry inference and phasing information is not needed for fully powered association statistics in admixed populations.

Goodness-of-fit tests and likelihood-ratio tests

Consider a 2 x 2 contingency table with ref/var counts 49/1 in cases and 25/25 in controls, assuming haploid samples. The Armitage Trend Test, a χ^2^(1dof) goodness-of-fit test defined as the number of samples times the squared correlation between genotype and phenotype, yields the value 29.94. On the other hand, a likelihood-ratio χ^2^(1dof) test which compares the causal model $p_{case}=0.95,p_{control}=0.50$ to the null model $p=0.74$ yields the value 35.49.

The two tests are asymptotically equivalent, but the approximation in the goodness-of-fit test can sometime yield a substantial deviation (see e.g. Wigginton et al. 2005 Am J Hum Genet). The likelihood-ratio test is the correct test, but the goodness-of-fit test can deviate from the correct statistic in either direction.

Adjustment of the scores to account for differences in imputation error rates in African and European segments

Let s be a causal SNP with odds ratio R and p be the maximum likelihood estimate frequency of the 0 allele. For notational simplicity we assume a haploid model but this is easily extended to the diploid case. When the SNP s is imputed, denote by p’ the maximum likelihood estimate of allele frequency of s from the imputed data. We can compute the standard r^2^ measure between the typed and imputed genotypes as follows. If we denote by p00, the number of times we see allele 0 imputed as 0, then

Let be the probability of imputing allele 0 given the true allele 0(1). It follows that:

(1)

Given that SNP s is causal with odds ratio R, then the difference in allele frequencies between cases and controls can be written as : where is the frequency in cases (controls). For simplicity we assume that the imputed allele frequencies are accurate (and ). We write the difference in allele frequencies between cases and controls in imputed data as:

Assuming we want to fit an additive model with odds ratio R’ over the imputed data, it follows that:

(2)

From (1) and (2) it follows that . It follows that R’ is a function of the true R, the allele frequency, and the imputation quality:

.

Note that when the imputation is perfect () then, as desired, the above formula reduces to R’=R. When imputation is inaccurate (), then the observed odds ratio on imputed data converges to 1.

We note that the observed odds ratio over imputed data depends on the allele frequency, the imputation quality and the true underlying odds ratio. Thus, for different populations, with different imputation qualities and different allele frequencies we expect to observe heterogeneity in the observed odds ratios due to different imputation qualities.

Unfortunately we do not know the true genotypes, and thus cannot compute the correlation between the true and imputed genotypes. However, reliable estimates for this correlation have been proposed; here we chose to use MACHestimates shown to produce robust estimates of imputation quality. To estimate ancestry specific imputation error rates, we restrict the computation to segments containing both alleles from that ancestry. Given that imputation accuracies are estimated directly from the data, depend on the term R and the allele frequencies. The causal likelihood computation of the MIX score, is maximized over the ‘true’ underlying odds ratio R, however, in the likelihood computation we replace the allelic odds ratio with the population specific odds ratios. In a similar fashion, we update the causal likelihood computation in the SNP1 score, except the ancestry odds ratio that is not used.
